# PLI Cancellation in ECG Signal Based on Adaptive Filter by Using Wiener-Hopf Equation for Providing Initial Condition

**DOI:** 10.1155/2014/471409

**Published:** 2014-07-23

**Authors:** Anchalee Manosueb, Jeerasuda Koseeyaporn, Paramote Wardkein

**Affiliations:** Telecommunications Engineering Department, Faculty of Engineering, King Mongkut's Institute of Technology Ladkrabang, Chalongkrung Road, Ladkrabang, Bangkok 10520, Thailand

## Abstract

This paper presents a technique for finding the optimal initial weight for adaptive filter by using difference equation. The obtained analytical response of the system identifies the appropriate weights for the system and shows that the MSE depends on the initial weight. The proposed technique is applied to eliminate the known frequency power line interference (PLI) signal in the electrocardiogram (ECG) signal. The PLI signal is considered as a combination of cosine and sine signals. The adaptive filter, therefore, attempts to adjust the amplitude of cosine and sine signals to synthesize a reference signal very similar to the contaminated PLI signal. To compare the potential of the proposed technique to other techniques, the system is simulated by using the Matlab program and the TMS320C6713 digital board. The simulation results demonstrate that the proposed technique enables the system to eliminate the PLI signal with the fastest time and gains the superior results of the recovered ECG signal.

## 1. Introduction

Nowadays, the number of patients with cardiac disorders continuously increases. Certain inappropriate habits in daily life, such as low physical activity, can lead to the risk factors for heart disease. Moreover, the improper eating habits might cause hyperlipidemia, incurrent disease of hypertension, and diabetes. There are several forms of cardiac disorder, for instance, coronary heart disease, enlarged heart, valvular heart disease, and myocardial disease due to myocardial infarction. The detection of these symptoms commonly relies on medical professionals to diagnose various factors to identify the exact abnormality of the patient's heart. The electrocardiogram (ECG) signal is a periodic waveform, which represents electrical occurrences during one heartbeat. Therefore, the interpretation of the ECG waveform is one basic technique that is used in the diagnosis of cardiac disorders. If an abnormality exists in the ECG waveform, it implies that the heart is also functioning abnormally; then, an in-depth diagnosis should be conducted. The ECG signal is a voltage signal that occurs in cardiac myocyte; it results from the exchange of the mineral concentration, such as sodium ions outside and potassium ions inside the cell. The amplitude of the ECG signal is typically very small (less than 20 mV). In the measurement of the ECG signal, the electrical device, which consists of several circuits, is used to acquire the signal. Although digital signal processing is applied through the process of signal acquisition [1–4], unfortunately, the acquired ECG waveform is still contaminated by the power line interference (PLI). If the PLI's amplitude is greater than 1% of the ECG's amplitude, it may affect the diagnosis of the medical professionals.

According to the mentioned information, the PLI signal usually occurs during the processes of ECG signal acquiring. It distorts the ECG waveform and causes the difficulties in the diagnosis procedure. By reviewing the existing literature, various methods for cancelling the PLI signal had been proposed. For example, the method which was proposed by Levkov et al. [6] generates the reference PLI signal by delaying the contaminated signal. This means that the reference PLI signal and the contaminated PLI signal have equal frequency and amplitude, but different phase. Then, the PLI cancellation is achieved by adding the generated reference PLI signal to the input signal. However, this approach will gain the dissatisfying results when the frequency, phase, or amplitude of the PLI signal varies over the time.

Using an adaptive filter is another effective method to eliminate the PLI signal. The adaptive filter has been mostly applied in signal processing [7–9] such as prediction, system identification, equalization, demodulation, and noise cancellation as echo cancellation [10–12], the denoising of heart sound [13–15], and PLI elimination [16–18]. For example, Widrow et al. [19] applied the least mean square (LMS) algorithm to adaptive filter to cancel the 60 Hz PLI signal. In this system, the amplitude and phase of the reference signal are determined by using two adapted weights, which will be adapted until the amplitude and phase are optimally close to those of the PLI signal. After that, So [20] presented the method called ASIC (adaptive sinusoidal interference canceller). In this method, the PLI signal and the input signal of the adaptive filter are defined in the form of sinusoidal signals. Two adapted weights, which correspond to the amplitude and phase parameters of the reference signal, are adapted to obtain the best reference PLI signal. Although the amplitude and phase parameters of the reference PLI signal are directly adapted, the ASIC technique does not provide the good result in the case of the time varying PLI signal. This occurs because the adaptation of amplitude and phase in the ASIC technique is not independent.

In 2008, Kanachareon [21] proposed a method for PLI cancellation where the PLI signal and the reference PLI signal are defined as the summation of cosine and sine signals. The reference PLI signal of the system is generated by adapting the amplitude of cosine and sine signals. Based on this approach, even the PLI signal is time variant; it can be eliminated. However, this PLI cancellation system may not work effectively, if the initial condition is not proper. In other words, the drawback of this technique is that the performance of the adaptive system depends on the proper initial condition. Later in 2009, Koseeyaporn et al. [5] proposed an enhanced adaptive algorithm for PLI cancellation in ECG signal where the two first samples are employed to find the best initial value of amplitude and phase of the reference PLI signal. With the defined initial conditions, this proposed technique can quickly eliminate the PLI signal. However, if the difference between the amplitudes of the two used samples is more than the average amplitude of the ECG signal, it will cause the improper initial conditions.

In this paper, a technique to obtain the initial weights for LMS based on the adaptive algorithm is presented. The initial weights are determined by representing the adapted weight equation in the form of the difference equation. With this technique, it can be applied for PLI cancellation. The paper is organized as follows: a technique for finding the optimal initial weight and the method for defining some variables are described in Section 2. The results of computer simulation are given in Section 3. Finally, Section 4 is the conclusion.

## 2. Method

### 2.1. The Proposed Method Based on Adaptive Filter for Eliminating the PLI Signal

**Figure 1:**
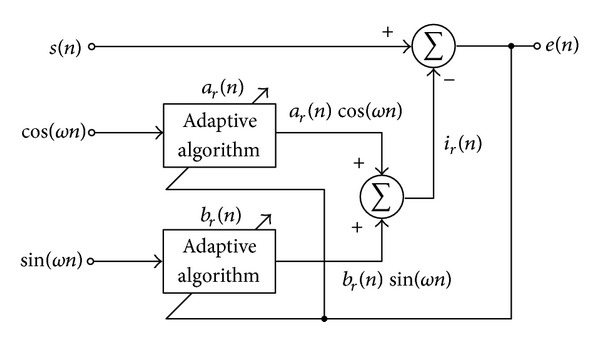
Block diagram of the proposed adaptive filter for eliminating PLI signal.

The proposed technique for eliminating the PLI signal, which corrupts in the ECG signal, is based on the adaptive algorithm. The block diagram of this technique is shown in Figure 1 [22]. The recovered ECG signal *e*(*n*) and the corrupted ECG signal *s*(*n*) can be written as

(1) $$\begin{array}{c} e\left(n\right)=s\left(n\right)-i_{r}\left(n\right), \end{array}$$

(2) $$\begin{array}{c} s\left(n\right)=d\left(n\right)+i\left(n\right), \end{array}$$

where *i*_*r*_(*n*) is the reference PLI signal which is generated by the system, *d*(*n*) is the original ECG signal, and *i*(*n*) is the contaminated PLI signal which is assumed to be a single frequency sinusoid. For the PLI signal, it is expressed in the following equation:

(3) $$\begin{array}{c} i\left(n\right)=\alpha \left(n\right)\cos\left(\omega n+\phi \left(n\right)\right), \end{array}$$

where *α*(*n*) and *ϕ*(*n*) are unknown amplitude and phase, respectively. Mathematically, (3) can be rewritten in the following form:

(4) $$\begin{array}{c} i\left(n\right)=a\left(n\right)\cos\left(\omega n\right)+b\left(n\right)\sin\left(\omega n\right), \end{array}$$

where *a*(*n*) and *b*(*n*) are the amplitude parameters of the cosine and sine terms, respectively. The relationship between the variables of (3) and (4) is given by

(5) $$\begin{array}{c} \left(n\right)=\sqrt{a^{2}\left(n\right)+b^{2}\left(n\right)}\\ \phi \left(n\right)=\text{ta}{\text{n}}^{-1}\left(\frac{-b\left(n\right)}{a\left(n\right)}\right). \end{array}$$

By using (4), (2) is rewritten to be

(6) $$\begin{array}{c} s\left(n\right)=d\left(n\right)+a\left(n\right)\cos\left(\omega n\right)+b\left(n\right)\sin\left(\omega n\right). \end{array}$$

And the reference signal of the system is rewritten as

(7) $$\begin{array}{c} i_{r}\left(n\right)=a_{r}\left(n\right)\cos\left(\omega n\right)+b_{r}\left(n\right)\sin\left(\omega n\right). \end{array}$$

Let *a*_*r*_(*n*) and *b*_*r*_(*n*) be the adaptive weights of cos⁡(*ωn*) and sin(*ωn*), respectively. Thus, the recovered signal as shown in (1) is given by

(8) e(n)=d(n)+a(n)cos⁡(ωn)+b(n)sin(ωn)−ar(n)cos⁡(ωn)−br(n)sin(ωn).

From (8), if the adaptive filter can adjust the adaptive weights *a*_*r*_(*n*) and *b*_*r*_(*n*), respectively, to *a*(*n*) and *b*(*n*), the recovered signal *e*(*n*), therefore, is *d*(*n*). The parameters *a*_*r*_(*n*) and *b*_*r*_(*n*) are adapted according to the following equations:

(9) ar(n+1)=ar(n)+μa∂e2(n)∂ar(n)=ar(n)+2μae(n)cos⁡(ωn)

(10) br(n+1)=br(n)−μb∂e2(n)∂br(n)=br(n)+2μbe(n)sin(ωn),

where *μ*_*a*_, *μ*_*b*_ are the step size values of the adaptive algorithm, which is 0 < *μ*_*a*_, *μ*_*b*_ < 1.

### 2.2. A Technique for Finding the Optimal Initial Weight

**Figure 2:**
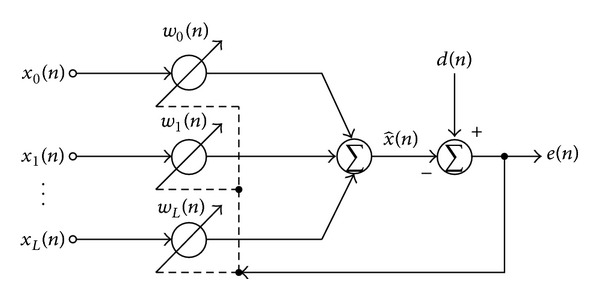
Block diagram of adaptive linear combination filter.

This technique realizes an adaptive filter as the linear combination filter, which is depicted in Figure 2. The error signal of the adaptive filter *e*(*n*) and the reference signal $\hat{x}\left(n\right)$ of the system are given by

(11) $$\begin{array}{c} e\left(n\right)=d\left(n\right)-\hat{x}\left(n\right),\\ \hat{x}\left(n\right)=X^{T}\left(n\right)w\left(n\right), \end{array}$$

where *d*(*n*) is the desired signal, **X**(*n*) is the input column vector of the adaptive filter, and **w**(*n*) is the adapted weight column vector. The weight vector is adapted by

(12) $$\begin{array}{c} w\left(n+1\right)=w\left(n\right)-\mu \frac{\partial \xi \left(w\left(n\right)\right)}{\partial w\left(n\right)}, \end{array}$$

where *μ* is the step size value, which is 0 < *μ* < 1, and *ξ*(**w**(*n*)) is the mean square error that is determined by

(13) ξ(w(n))=E[e2(n)]=E[(d(n)−x^(n))2]=E[(d(n)−XT(n)w(n))2]=E[d2(n)]−2rdxTw(n)+wT(n)Rxxw(n).

Let E[·] be the expectation operation, **r**_*dx*_ the cross-correlation between the desired signal and the input signal, and **R**_*xx*_ the autocorrelation of the input signal. By replacing (13) into (12), it yields

(14) w(n+1)=w(n) −μ∂(E[d2(n)]−2wT(n)rdx+wT(n)Rxxw(n))∂w(n)=w(n)−μ[−2rdx+2Rxxw(n)]=w(n)+2μrdx−2μRxxw(n).

When the system reaches the convergence state, **w**(*n* + 1) = **w**(*n*) converges to **w**_*o*_, which is the optimal adapted weight, and can be defined by

(15) $$\begin{array}{c} w_{o}=w_{o}+2\mu r_{dx}-2\mu R_{xx}w_{o}=R_{xx}^{-1}r_{dx}. \end{array}$$

This equation is called the Wiener-Hopf equation. By considering (14), it can be rearranged in the form of the difference equation as

(16) $$\begin{array}{c} w\left(n\right)=\left[I-2\mu R_{xx}\right]w\left(n-1\right)+2\mu r_{dx}\\ w\left(n\right)-\left[I-2\mu R_{xx}\right]w\left(n-1\right)=2\mu r_{dx}. \end{array}$$

In the form of difference equation as given by (16), the natural response equation is found to be

(17) $$\begin{array}{c} w_{n}\left(n\right)-\left[I-2\mu R_{xx}\right]w_{n}\left(n-1\right)=0. \end{array}$$

Let **w**_*n*_(*n*) = **r** ^*n*^ **C**, where **C** is a constant vector. By solving (17), it is found that

(18) $$\begin{array}{c} r^{n}C-\left[I-2\mu R_{xx}\right]r^{n-1}C=0,\\ r=\left[I-2\mu R_{xx}\right]. \end{array}$$

Then, the natural response is

(19) $$\begin{array}{c} w_{n}\left(n\right)={\left[I-2\mu R_{xx}\right]}^{n}C. \end{array}$$

In addition, the forced response equation, which is considered from (16), is

(20) $$\begin{array}{c} w_{f}\left(n\right)-w_{f}\left(n-1\right)\left[I-2\mu R_{xx}\right]=2\mu r_{dx}. \end{array}$$

In this paper, it is assumed that **R**_*xx*_ and **r**_*dx*_ are changed very slowly when compared with *n*; therefore, these two parameters are considered as constants. By defining **w**_*f*_(*n*) = **A**, the forced response is solved as follows:

(21) $$\begin{array}{c} A-\left[I-2\mu R_{xx}\right]A=2\mu r_{dx}\\ A=R_{xx}^{-1}r_{dx}. \end{array}$$

Hence, the impulse response of the adaptive filter is

(22) $$\begin{array}{c} w\left(n\right)=w_{n}\left(n\right)+w_{f}\left(n\right)={\left[I-2\mu R_{xx}\right]}^{n}C+R_{xx}^{-1}r_{dx}. \end{array}$$

From (22), by defining *n* = 0, the constant vector **C** is found to be

(23) $$\begin{array}{c} w\left(0\right)={\left[I-2\mu R_{xx}\right]}^{0}C+R_{xx}^{-1}r_{dx}=C+R_{xx}^{-1}r_{dx},\\ C=w\left(0\right)-R_{xx}^{-1}r_{dx}. \end{array}$$

By replacing (23) into (22), it yields

(24) $$\begin{array}{c} w\left(n\right)={\left[I-2\mu R_{xx}\right]}^{n}\left[w\left(0\right)-R_{xx}^{-1}r_{dx}\right]+R_{xx}^{-1}r_{dx}, \end{array}$$

where **w**(0) is the initial weight of the adaptive filter. By letting **w**(0) = **R**_*xx*_^−1^ **r**_*dx*_ and replacing it into (24), the impulse response of the system will be

(25) $$\begin{array}{c} w\left(n\right)={\left[I-2\mu R_{xx}\right]}^{n}\left[R_{xx}^{-1}r_{dx}-R_{xx}^{-1}r_{dx}\right]+R_{xx}^{-1}r_{dx}=R_{xx}^{-1}r_{dx}. \end{array}$$

By considering (25), it is seen that the adaptive filter has converted to convergent state at any *n*.

### 2.3. The Performance of the System in Terms of MSE Related to the Initial Weight

From the MSE given in (13) and the impulse response of adaptive filter given in (24), it is found that

(26) ξ(w(n))=E[d2(n)] −2rdxT{[I−2μRxx]n[w(0)−Rxx−1rdx]+Rxx−1rdx} +{[I−2μRxx]n[w(0)−Rxx−1rdx]+Rxx−1rdx}T ×Rxx{[I−2μRxx]n[w(0)−Rxx−1rdx]+Rxx−1rdx  }ξ(w(n))=E[d2(n)]+{[I−2μRxx]n[w(0)−Rxx−1rdx]}T ×Rxx{[I−2μRxx]n[w(0)−Rxx−1rdx]} −rdxTRxx−1rdx.

**Figure 3:**
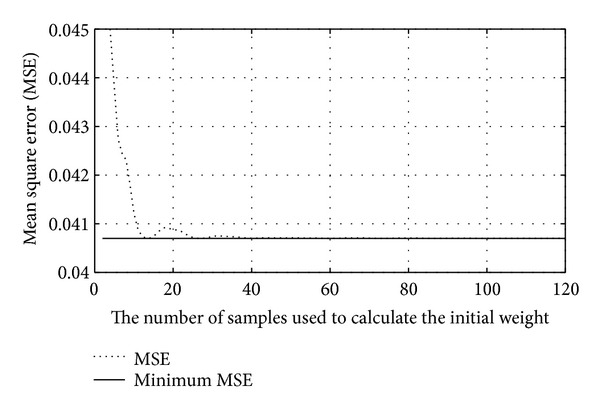
MSE of the system versus the number of samples employed in the initial weight calculation.

From (26), it implies that the initial weight **w**(0) affects the MSE of the system. The minimum MSE will be achieved, if the second term of (26) is close to zero.  Figure 3 illustrates the MSE of the system versus the number of samples, which is used to determine the initial weight (dashed line). It is seen that the more samples, the less MSE of the system. From Figure 3, when the number of samples is more than 30 samples, the minimum MSE is approximately achieved.

### 2.4. The Method for Finding a Proper Number of Samples to Calculate the Optimum Initial Weight

It is well known that the initial weight is an important factor for the convergence rate of the adaptive filter. In addition, as shown in the previous subsection, the number of samples for calculating the initial weight is related to the MSE of the system, which indicates the efficiency of the adaptive filter. It, thus, gives rise to a question regarding how to find a proper number of samples for determining the initial weight.

In this paper, the number of samples used to calculate the initial weight will be determined by using two criteria as the number of the mathematical operations and the acceptable value of the MSE.

The number of mathematical operations, multiplication and addition, for calculating the initial weight, is shown in (27) and (28), respectively.

Consider the following:

(27) $$\begin{array}{c} \text{Number}\;\text{of}\;\text{ Multipliers }=\left(N+N^{2}\right)\left(m+1\right)+N^{2}+\frac{N^{3}}{2}, \end{array}$$

(28) $$\begin{array}{c} \text{Number}\;\text{of}\;\text{ Adders }=\left(N+N^{2}\right)\left(m-1\right)+N^{2}-N+\frac{N^{3}}{2}, \end{array}$$

where *N* is the number of input signals of the adaptive filter, which is 2 in this case (cosine and sine signals), and *m* is the number of samples for calculating.

**Figure 4:**
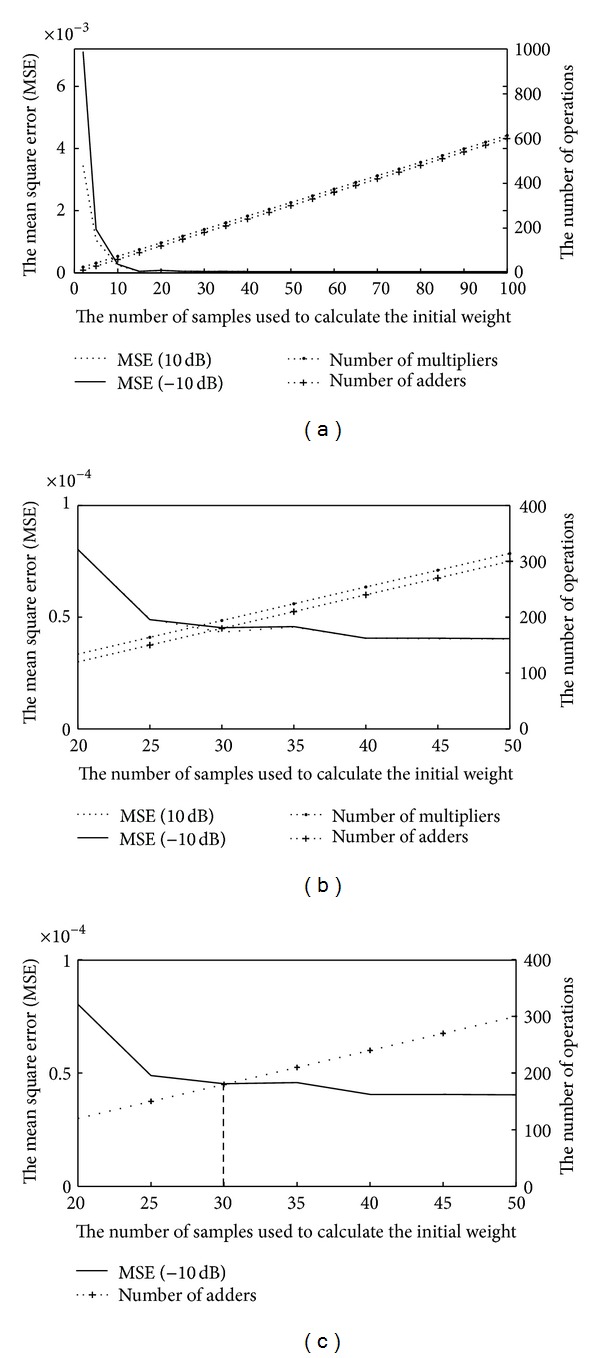
The relationship between the MSE and the number of mathematical operations versus the number of samples used in calculating: (a) with no constraint, (b) with acceptable requirement, and (c) the intersection of the graph for finding the number of samples.

In this study, the intersection between the graph of the MSE of the system and the graph of the number of operations (multiplier and adder) is used to determine the proper number of samples used for calculating the initial weight. From Figure 4(a), it is the relationship between the MSE and the number of mathematical operations versus the number of samples used in calculating with no constraint. To determine the proper number of samples, the graphs of the MSE at the SNR = 10 dB (general case) and the SNR = −10 dB (the worst case) are considered. In this figure, there are 4 intersection points which may hardly be observed. For Figure 4(b), the graph is obtained by setting the conditions for the MSE to be less than 0.0001 and for the number of mathematical operations (multiplier and adder) to be 400. The number of samples is defined from the intersection point, which is the largest one. It is obtained from the intersection point between the graph of the MSE at the SNR = 10 dB and the graph of the number of adder operations. It is clearly illustrated in Figure 4(c) that the proper number of samples is 30 samples. The selected number of samples, thus, is 30. After that, the initial weight is shown as follows:

(29) d=[d(1)d(2)⋯d(30)];  input  signalx1=[x1(1)x1(2)⋯x1(30)]T;  cos⁡(ωn)x2=[x2(1)x2(2)⋯x2(30)]T;  sin(ωn)rdx=E[dx1dx2]TRxx=E[x1Tx1x1Tx2x2Tx1x2Tx2]wo=Rxx−1rdx.

## 3. The Simulation Results

In this section, the simulation results are presented. The proposed technique and two other techniques for determining initial weight, which are the technique of using random initial and the technique proposed by Koseeyaporn et al. [5], are used to compare the performance of the adaptive filter. The simulation results will be separated into two parts as follows.

### 3.1. The Simulation Results of Using the Matlab Program

**Figure 5:**
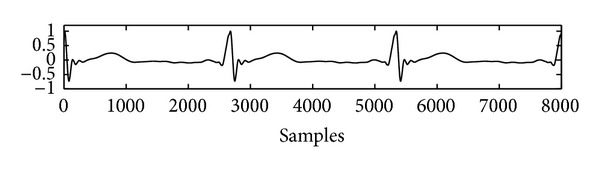
The ECG signal employed in the simulation.

**Figure 6:**
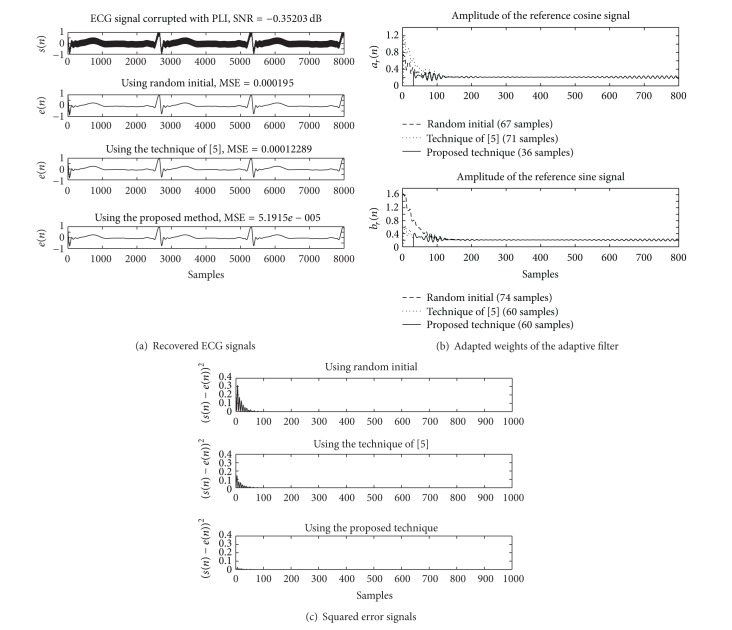
Comparing simulation results of using the Matlab program for SNR = −0.35 dB. (a) Contaminated ECG signal and recovered ECG signals. (b) Adapted weights of the cosine and sine signals. (c) Squared error signals.

**Figure 7:**
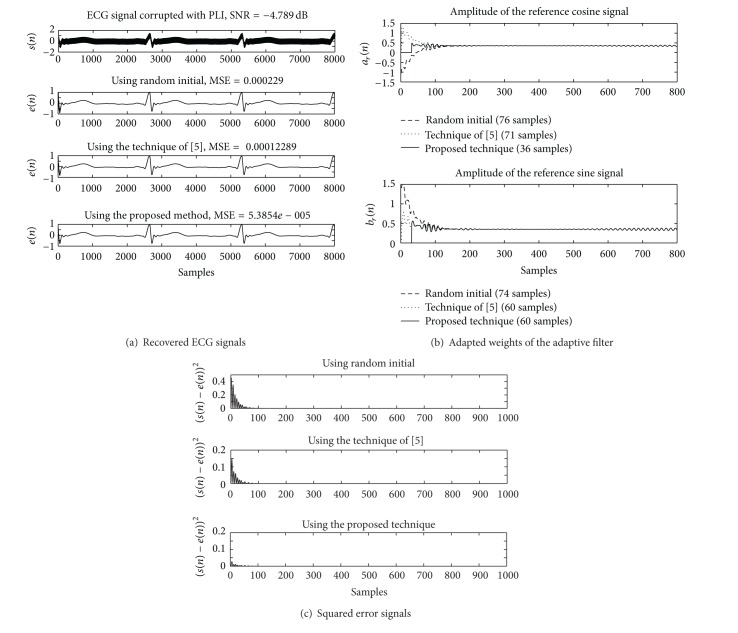
Comparing simulation results of using the Matlab program for SNR = −4.79 dB. (a) Contaminated ECG signal and recovered ECG signals. (b) Adapted weights of the cosine and sine signals. (c) Squared error signals.

**Figure 8:**
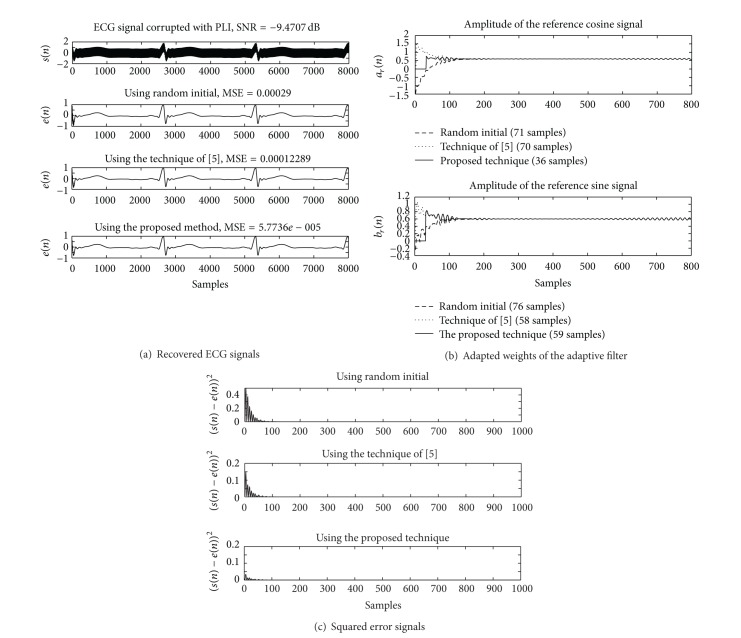
Comparing simulation results of using the Matlab program for SNR = −9.47 dB. (a) Contaminated ECG signal and recovered ECG signals. (b) Adapted weights of the cosine and sine signals. (c) Squared error signals.

The clean ECG signal employed in the simulation is illustrated in Figure 5. The adaptive filter is set up to eliminate the PLI signal which corrupts in the ECG signal, and the step size parameters defined as *μ*_*a*_ and *μ*_*b*_ are 0.032. The simulation results of using the Matlab program are depicted in Figures 6, 7, and 8 for the SNR at −0.35, −4.79, and −9.47 dB, respectively.

The recovered ECG signals as shown in Figure 6(a), which are obtained from three methods, are in different waveforms. The results show that the proposed method is superior to other methods, due to the lowest MSE. The number of samples to reach the convergence state in each technique, which is considered from the adapted weights of the adaptive filter, is given in Figure 6(b). In Figure 6(b), the numbers of samples used for the convergence of the reference cosine signal (*a*_*r*_(*n*)) are 67, 71, and 36 for using random initial, the technique of [5], and the proposed technique, respectively. The numbers of samples used for the convergence of the reference sine signal (*b*_*r*_(*n*)) are 74, 60, and 60 for using random initial, the technique of [5], and the proposed technique, respectively. The squared error signals obtained from the compared techniques are depicted in Figure 6(c). The results of the simulation for the SNR at −4.79 and −9.47 dB are demonstrated in Figures 7 and 8, respectively.

### 3.2. The Simulation Results of Using the TMS320C6713 Digital Board

**Figure 9:**
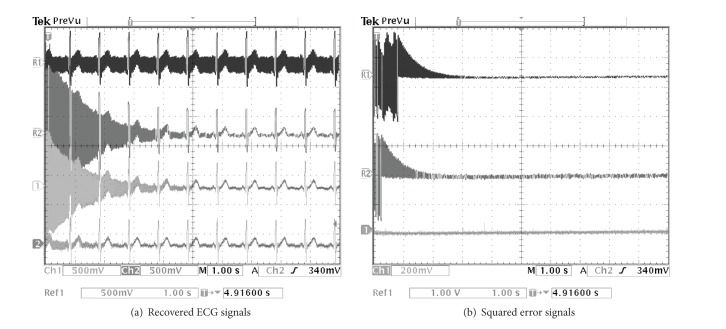
Comparing simulation results of using the TMS320C6713 digital board for SNR = −0.35 dB. (a) R1: contaminated ECG signal, R2: recovered ECG signal by using random initial, Ch1: recovered ECG signal of the technique of [5], and Ch2: recovered ECG signal of the proposed technique. (b) R1: squared error signal by using random initial, Ch1: squared error signal of the technique of [5], and Ch2: squared error signal of the proposed technique.

**Figure 10:**
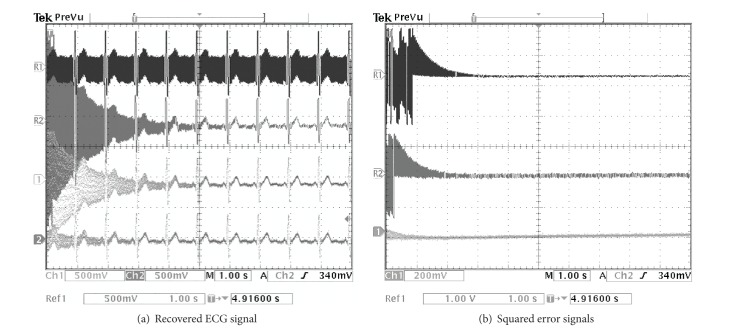
Comparing simulation results of using the TMS320C6713 digital board for SNR = −4.79 dB. (a) R1: contaminated ECG signal, R2: recovered ECG signal by using random initial, Ch1: recovered ECG signal of the technique of [5], and Ch2: recovered ECG signal of the proposed technique. (b) R1: squared error signal by using random initial, Ch1: squared error signal of the technique of [5], and Ch2: squared error signal of the proposed technique.

**Figure 11:**
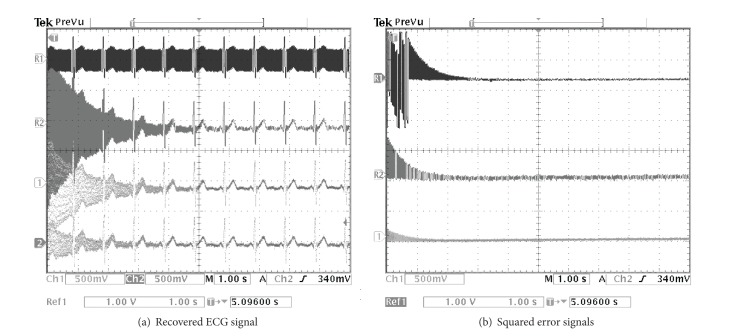
Comparing simulation results of using the TMS320C6713 digital board for SNR = −9.47 dB. (a) R1: contaminated ECG signal, R2: recovered ECG signal by using random initial, Ch1: recovered ECG signal of the technique of [5], and Ch2: recovered ECG signal of the proposed technique. (b) R1: squared error signal by using random initial, Ch1: squared error signal of the technique of [5], and Ch2: squared error signal of the proposed technique.

The PLI cancellation results from the TMS320C6713 digital board are depicted in Figures 9, 10, and 11 for the SNR of the input signal at −0.35, −4.79, and −9.47 dB, respectively. By considering from the beginning state of these results, it illustrates that the proposed method can eliminate the PLI signal with the fastest time which is accordant with the results of computer simulation.

## 4. Conclusion

A technique for finding the appropriate initial weights for the adaptive filter is proposed in this paper. The initial weights are determined by representing the adapted equation in the form of the difference equation. The derived analytical response identifies the appropriate weights for the system and shows that the MSE depends on the initial weights. The proposed technique aims to be applied for PLI cancellation in ECG signal. The results obtained from computer simulation by using the Matlab program and the TMS329C6713 digital board show that the adaptive filter based on the proposed technique for obtaining the proper initial weights can be applied in PLI cancellation and provides better performance than the compared techniques.
